# Mouse models of Anti-Aβ immunotherapies

**DOI:** 10.1186/s13024-025-00836-x

**Published:** 2025-05-13

**Authors:** Philip Pikus, R. Scott Turner, G. William Rebeck

**Affiliations:** 1 Department of Neuroscience, Georgetown University Medical Center, 3970 Reservoir Rd, NW, District of Columbia Washington, 20007 USA; 2 Interdisciplinary Program in Neuroscience, Georgetown University, 3970 Reservoir Rd, NW, District of Columbia Washington, 20007 USA; 3 Department of Neurology, Georgetown University Medical Center, 3800 Reservoir Rd, NW, District of Columbia Washington, 20007 USA

**Keywords:** Alzheimer’s disease, Amyloid-beta, Immunotherapy, Mouse models, Microglia, Cerebral amyloid angiopathy, Blood brain barrier, ARIA, Neuronal dysfunction

## Abstract

**Background:**

The development of anti-amyloid-beta (Aβ) immunotherapies as the first disease modifying therapy for Alzheimer’s Disease (AD) is a breakthrough of basic research and translational science.

**Main text:**

Genetically modified mouse models developed to study AD neuropathology and physiology were used for the discovery of Aβ immunotherapies and helped ultimately propel therapies to FDA approval. Nonetheless, the combination of modest efficacy and significant rates of an adverse side effect (amyloid related imaging abnormalities, ARIA), has prompted reverse translational research in these same mouse models to better understand the mechanism of the therapies.

**Conclusion:**

This review considers the use of these mouse models in understanding the mechanisms of Aβ clearance, cerebral amyloid angiopathy (CAA), blood brain barrier breakdown, neuroinflammation, and neuronal dysfunction in response to Aβ immunotherapy.

## Background

The development and subsequent FDA approval of passive immunotherapies to treat Alzheimer’s Disease (AD) is a major research advance and a source of hope for patients, caregivers, and loved ones. The monoclonal antibodies directed against the amyloid-beta (Aβ) peptide are the first disease modifying therapies for AD [1]. The approved immunotherapies, lecanemab and donanemab, effectively clear amyloid in the brains of AD patients and slow the rate of cognitive decline. This demonstration of therapeutic effects of Aβ immunotherapies supports the long-standing amyloid cascade hypothesis, developed largely from early genetic studies of AD [2].

One common side effect of Aβ immunotherapies is amyloid-related imaging abnormalities (ARIA), which occur in up to a third of patients receiving these drugs [3]. ARIA is classified into two categories - associated with edema (ARIA-E) reflecting fluid extravasation across a leaky blood brain barrier, or associated with hemorrhage (or hemoglobin or hemosiderin) (ARIA-H), reflecting leakage of blood products into the CNS [4]. Major risk factors for ARIA are the *APOE4* genotype, cerebral amyloid angiopathy (CAA), and high baseline amyloid burden [5]. Spontaneous forms of ARIA (in the absence of anti-amyloid therapies) can occur in humans due to the presence of CAA, as hemorrhages or as inflammation [6], which may be related to endogenous anti-amyloid antibodies [7]. This review highlights the importance and methods of reverse translational research to understand anti-Aβ immunotherapy and ARIA.

Over the past two decades, mouse models have been used to define the usefulness of these antibodies and to understand the underlying mechanisms of their actions. They incorporate different components of AD: Aβ plaques, gliosis, CAA, and tau pathologies. Although Aβ immunotherapies are now being used in humans, there are still major gaps in our understanding of the molecular mechanisms and side effects that come from these therapies. This review will summarize the research advances from these models and highlight how continued research in these models is necessary to address important new questions.

## Genetically modified mouse models

Many transgenic mouse models have been used during the development and analysis of Aβ immunotherapies. Key features of some of the main amyloid models that have been used are described here and listed in Table 1.

The PDAPP mice [8] overexpress the human beta-amyloid precursor protein (APP) with the Indiana (V717F) mutation, with expression driven by the PDGF-β promoter. The mutation at the 717 residue shifts Aβ production from Aβ40 to Aβ42, which results in increased parenchymal amyloidosis [8]. These mice develop amyloid plaques by about six months-of-age and CAA later, with extensive deposition observed at 22 months-of-age; the delayed presence of CAA may be due to the high Aβ42/40 ratio [9]. The PDAPP mice were the first used in the study of immunization against Aβ [10].

The 5XFAD mice [11] have five familial AD mutations: three in APP: the Swedish (K670N/M671L), Florida (I716V), and London (V717I) mutations; and two in presenilin-1 (PS1): the M146L and L286V mutations. These mutations are driven by the *Thy1* promoter [11]. There are two major lines of 5XFAD, one (#6799) that accumulates plaques as early as 1.5 months-of-age [11], and another (#7031) that accumulates plaques at about 4 months of age [12]. There is an increased Aβ42/40 ratio and little or no CAA in either line [13]. Both 5XFAD lines have been crossed with human APOE knock-in mice (*APOE2*, *APOE3*, and *APOE4*) [14] to generate EFAD mice for the study of *APOE* genotype in amyloidosis [12, 15]. When the 7031 line is crossed with human APOE4, there is a significant increase in CAA in mice aged between 8 and 10 months [12].

The Tg2576 mice [16] overexpress a mutant form of APP containing the K670/M671 mutation driven by the hamster prion protein promoter [16]. These mice develop significant amyloid plaques between 11 and 13 months of age [17]. They have high Aβ production but not an altered Aβ42/40 ratio [17]. In addition to increases in Aβ42, these mice exhibit a five-fold increase in Aβ40 levels by 10 months-of-age, with a corresponding increase in CAA [18].

The 3xTg mice [19] have three mutations that are associated with AD: M146V in PS1, K670/M671 in APP, and P301L in MAPT (microtubule-associated protein tau) [19]. The expression of these transgenes is regulated by the mouse Thy1.2 promoter [17]. They develop Aβ plaques at approximately six months-of-age with a high Aβ42/40 ratio [13]. While CAA has not been demonstrated in these mice, they have exhibited astrocytic hyperactivity that damages endothelial cells, as well as significant deficits in smaller vessels [20, 21]. These changes occurred before plaque deposition and were generally confined to the microvasculature and not larger vessels [21].

The APP/PS1 mice combine the Swedish APP mutation with a PS1 L166P mutation [22]. Both mutations are driven by the Thy1 promoter [22]. Similar to the Tg2576 mice, both Aβ42 and Aβ40 levels increase, but the Aβ42/40 ratio decreases in the brain, differentiating it from other AD models [23]. Amyloid plaques form around three to four months of age, while CAA has not been prominent [24].

The APP23 mice [25] have the Swedish double mutation causing a seven-fold increase in APP expression under the control of the Thy1 promoter [25], resulting in an increase in both Aβ42 and Aβ40. However, there is a more rapid increase in Aβ42 resulting in an increased Aβ42/40 ratio [26]. Amyloid plaques develop by about six months-of-age [25] while significant CAA was observed in APP23 mice beginning at around 19 months-of-age [27].

The hAPP (J20) mice [28] have both the APP Swedish and Indiana mutations driven by the PDGF-β promoter [28]. These mice exhibit synaptic deficits, neuronal loss, and neuroinflammation before signs of plaque deposition [29, 30]. Plaques develop at around 7-months-of-age [30]. No significant CAA deposition has been reported in this model [30].

**Table 1:** Characteristics of genetically modified mouse models that were more commonly used in the study of anti-Aβ immunotherapies

Mouse line	Age of parenchymal Aβ deposition	Amount of CAA	Age of CAA Deposition	Crosses with other lines described in this review	Commercial availability
PDAPP	6–9 months [8]	Moderate [9]	Extensive CAA at 22 months [9]	GFP-fluorescent microglia (expressed on Cx3Cr1 promoter) [31]	
5XFAD B6SJL 5XFAD C57BL/6J	1.5 months [11] 4 months [12]	Very Low [13] Low [12]	Some CAA in E4FAD [15] at 8–10 months [12]	GFP-microglia, APOE2/3/4 [15] APOE4 [12]	Jackson Laboratory Stock #034840 Jackson Laboratory Stock #034848
Tg2576	11–13 months [17]	High [18]	By 19 months most pial arteries are affected [18]	None discussed here	Taconic Biosciences Stock #1349
3xTg	6 months [13]	Low [21]	~ 12 months [21]	None discussed here	Jackson Laboratory Stock #034830
APP/PS1	3–4 months [23]	Not prominent [24]	Not prominent [24]	None discussed here	Jackson Laboratory Stock #034832
APP23	6 months [25]	Moderate/high with aging [27]	~ 19 months [27]	None discussed here	Jackson Laboratory Stock #030504
hAPP	7 months [30]	None reported [30]		None discussed here	Jackson Laboratory Stock #034836

## Anti-Aβ monoclonal antibodies

Many monoclonal antibodies have been developed to target Aβ in these mouse models, including several that have been humanized and used in clinical trials and clinical settings. The key features of the antibodies discussed in this review are listed here, and the antibodies that are discussed in most detail are listed in Table 2. The earliest mouse studies used active immunotherapy, with mice injected with Aβ42 [10]. Subsequent studies developed passive immunotherapy as a mechanism to clear Aβ plaques and treat AD.

Many of the successful antibodies examined target N-terminal epitopes of Aβ. Antibody 10D5 is an IgG1 isotype monoclonal antibody targeting Aβ amino acids 3–7 [32]. Antibody 3D6 has been developed as both an IgG2b and IgG1 isotype [33, 34], and is a monoclonal antibody targeting Aβ amino acids 1–5 [35]; its ability to clear plaques led to its humanized version of bapineuzumab. PFA-1 is an IgG2 isotype monoclonal antibody that recognizes Aβ amino acids 3–6, showing a high affinity for fibrils and protofibrils [36]. Antibody 5C8H5 is derived from 4Aβ1–15 and antibody 3F5 is directed against amino acids Aβ 1–11 [37, 38]. A β1 mouse monoclonal antibody has also been used which targets Aβ amino acids 3–6 [39]. Finally, aducanumab was the first anti-Aβ immunotherapy to achieve FDA approval [40, 41]; its murine version is an IgG2a isotype that binds Aβ amino acids 3–7 [42].

Other anti-Aβ antibodies have been used to target C-terminal domains. Antibody 21F12 targets Aβ amino acids 33–42 of in the C-terminus; it did not significantly bind plaques or trigger phagocytosis in ex vivo studies [32]. The 2286 antibody is an IgG1 isotype that recognizes Aβ amino acids 28–40 [43]. Antibody 2H6 is an IgG2b antibody that targets Aβ amino acids 33–40; it has also been studied in a de-glycosylated form [44].

The FDA-approved drug Donanemab is an IgG1 isotype monoclonal antibody directed against the N-terminal truncated and pyroglutamate (amino acid number 3) modified Aβ [45]. While Donanemab cleared plaques in preclinical studies [34], the pyroglutamate epitope appears later in transgenic mouse models compared with human AD patients, impacting the ability to translate preclinical experiments to clinical studies using this therapy [46].

The antibody Lecanemab does not target specific amino acids of Aβ, but soluble Aβ protofibrils [47]. Lecanemab was developed from mAb158, an IgG2a isotype antibody [48]. It successfully clears plaques [47] and is FDA-approved for treatment of AD.

Antibodies lacking the Fc portion (constant region) are known as Fab fragments, which generally fail to trigger an immune response, as the Fc portion of the antibody interacts with immune cells [49]. Thus, the Fc domain is an important component of microglia activation across CNS disorders [50]. Some studies have used 3D6 without the Fc portion in order to test the impact on triggering microglial-mediated phagocytosis [51]. Another approach is to use single-chain variable fragment (scFv) versions of these antibodies, essentially only including the variable regions of the antibody [52]. This smaller version of the antibodies allows for easier and potentially safer transport [52]. In this review this approach is addressed with scFv versions of 3D6 and mAb158 [53–55].

Finally, Trontinemab [56] is a modified version of the monoclonal antibody gantenerumab (targeting the mid-domain of Aβ) with the human transferrin receptor 1 fused to it. This fusion antibody has been tested in non-human primates [56].

**Table 2:** Monoclonal antibodies directed against Aβ that are discussed in detail in this review

Antibody	Clinical/ Preclinical Names	Isotype	Epitope	Translation to Humans
Active Immunization	AN1792	N/A	N/A	Underwent clinical trials that were ultimately halted [57]
10D5	N/A	IgG1 [32]	Aβ amino acids 3–7	No
3D6	Bapineuzumab (humanized)	IgG1, IgG2b [33, 34]	Aβ amino acids 1–5	Underwent clinical trials
2286	N/A	IgG1 [43]	Aβ amino acids 28–40	No
Aducanumab	Aduhelm (brand name)	IgG2a [42]	Aβ amino acids 3–7	Yes
Lecanemab	Leqembi (brand name), BAN2401, mAb158	IgG2a [48]	Large, soluble protofibrils	Yes
Donanemab	Kisunla (brand name), LY3002813	IgG1 [34]	Aβ(p3-42), pyroglutamate	Yes

## Clearance of Aβ plaques

The primary neuropathological endpoint in the study of anti-Aβ immunotherapies is the decrease in levels of parenchymal Aβ plaques. The earliest studies were of active immunotherapy, with 11 month old PDAPP mice immunized with Aβ42 chronically over an 11 month treatment period [10]. There were significant and large decreases in Aβ levels in the brain both when measured by immunohistochemistry and ELISA, showing that active immunotherapy was capable of either removing existing amyloid plaques or preventing their initial deposition [10]. The approach of using active vaccination against Aβ was also studied as a preventative approach against AD, with six month-old Tg2576 mice treated with nine immunizations [58]. These treatments significantly decreased plaque load and soluble Aβ levels even months following the final immunization, at 15 months-of-age [58]. However, passive immunotherapy is the approach that has ultimately generated more translational studies.

### Antibody epitopes and forms

Early studies of passive immunotherapy focused on the movement of antibody to the brain following peripheral injection. A single tracer dose of 3D6 was administered either intraperitoneally (IP) and intravenously (IV) dosing to hAPP mice, and levels of both serum and brain antibody were measured [59]. While levels of 3D6 gradually declined in the serum post-injection, levels in the brain increased by three days (the first time point measured) and for as long as 27 days, with higher levels of antibody correlating with more Aβ-rich regions, such as the hippocampus. Most of the results focused on the IV injection, and no direct comparison between IP and IV dosing was reported [59]. Thus, peripherally administered antibodies not only reached the brain quickly but also remained in the brain for a sustained period following injection.

Other early studies of passive immunotherapy addressed which characteristics of the antibodies led to the most amyloid clearance. In general, these antibodies were administered peripherally from 3 to 40 mg/kg, mostly through IP injections but also sometimes through IV injections. In one study, 10D5 and 21F12, which bind to Aβ N and C-termini respectively, were administered IP to PDAPP mice [51]. Following six months of passive immunotherapy, immunofluorescence studies revealed that 10D5 cleared parenchymal Aβ plaques and ELISA revealed a significant reduction in total amyloid burden; 21F12 had a more modest effect [51]. These results suggested that antibodies targeting the Aβ N-terminus were more effective in amyloid clearance compared with those targeting the C-terminus. Another early study compared antibodies selectively targeting Aβ residue 42, residue 40 and residues 1–16. They found that while all three approaches prevented initial plaque deposition, only the antibody targeting the N-terminus 1-16 residues significantly cleared existing plaques [60]. In another comparison study, 3D6 (which binds the N-terminus) bound to plaques and cleared insoluble Aβ more effectively than gantenerumab or crenezumab, which both bind the mid-domain of Aβ [61]. Another monoclonal antibody targeting the N-terminus of Aβ, 3F5, also lowered plaque-load, promoted microglial phagocytosis of plaques, and attenuated neuronal cell death, when administered IP to APP/PS1 mice twice monthly for 3 months [37]. These preclinical studies contributed to the use of N-terminus targeting antibodies in early clinical trials.

The application of antibodies directly to the brains of mice allowed for the analysis of immediate effects of Aβ antibodies. Early studies using antibody 2286 in 19 month-old Tg2576 mice showed that when antibody was applied directly to the frontal cortex and hippocampus following stereotaxic surgery, there was significant Aβ clearance measured by immunostaining [62]. 10D5 and 3D6 were applied to the brains of both PDAPP and Tg2576 mice aged 20-months following craniotomy; two-photon microscopy allowed for in vivo visualization of amyloid deposits through application of either thioflavin S or immunofluorescent-tagged Aβ antibodies [63]. A single dose of 10D5 or 3D6 resulted in significant decreases in diffuse Aβ deposits within three days [63]. These studies showed that when antibodies have immediate access to the brain, they can efficiently clear plaques.

Removal of the Fc portion from 3D6 maintained a similar percent clearance of diffuse Aβ when administered directly to the brain [64]. These data suggested that in addition to Fc-mediated clearance, interaction of antibody with amyloid could be contributing to clearance in the absence of microglial phagocytosis [64]. In another study, antibody 10D5 was administered IP weekly for six months; plaque clearance occurred, with Aβ localized within microglia after cell sorting from unfixed ex vivo tissue [51]. When the Fc portion was removed, 10D5 was unable to trigger microglia-mediated phagocytosis and did not clear plaques, demonstrating a need of the Fc domain in this model [51]. More studies are needed to elucidate the relative contributions of Fc-mediated and Fc-independent Aβ clearance mechanisms. Based on these few studies, antibody directly injected into the brain may be able to clear Aβ via Fc-independent mechanisms, but the Fc portion may be important in the clearance of plaques upon peripheral administration.

Another approach to assess antibody clearance of plaques is to use single-chain variable fragment versions of these antibodies, both eliminating the Fc domain and developing a smaller antibody that is more easily transported to the brain. A single-chain variable fragment version of 3D6 (scFv-h3D6) effectively lowered intracellular Aβ after a single 100 *µ*g dose IP, five days post-treatment [53], while another study showed reduced levels of Aβ oligomers in the cortex following an 85 *µ*g IP dose of this antibody [65]. Each of these studies were performed in five-month-old 3xTg mice. These studies suggest that with this scFv, even a single dose of antibody delivered IP is capable of clearing Aβ in different forms. Further studies would be needed to determine whether this success is a property intrinsic to the single chain fragment or whether it is the smaller size of the antibody that is contributing to more efficient plaque clearance.

Trontinemab is a molecule developed by fusing gantenerumab with a monoclonal antibody against the human transferrin receptor 1 [56]. In non-human primates, trontinemab was found to more efficiently reach the brain and clear plaques compared with gantenerumab [56]. This facilitated transport into the brain has the potential to allow for lower doses of antibodies to be administered, potentially leading to fewer side effects.

These models demonstrated that peripherally administered antibodies have quick effects on Aβ clearance but that total clearance requires time and likely multiple treatments. In Tg2576 mice, passive immunotherapy with the monoclonal antibody 2286 (recognizing Aβ 28–40) showed different levels of changes in amyloid load and microglial activation after one, two, and three months of treatment [43]. While total concentrations of antibody 2286 in the brain increased quickly upon initiation of immunotherapy, and remained high over the three-month experimental period, there was only a slight decrease in Aβ protein deposition in the first month, primarily confined to more diffuse plaques. However, a 50% decrease in total parenchymal Aβ deposits in both the frontal cortex and hippocampus occurred between one and two months of treatment, as measured by Congo red staining [43]. The antibody RmAb158, along with a mutated version of this antibody with the Fc portion removed (RmAb158-scFv8D3), were tested for their ability to clear Aβ both following a single dose and multiple doses in APP mice. When five-month-old mice were treated with a single dose, there was no significant reduction in total Aβ1–42 levels in the cortex or hippocampus. However, in both conditions when three doses were administered and when antibody was administered chronically for nine weeks, there were significant decreases in Aβ levels [54]. Although antibody that is peripherally administered quickly accesses the brain, and antibody applied directly to the brain clears plaques within days, important questions remain regarding the timing of early plaque clearance by passive immunotherapies.

Passive immunotherapy has been shown to clear plaques of different morphologies. Antibody 12B4, an IgG2a antibody recognizing amino acids 3–7 of Aβ [66], was able to significantly clear both more diffuse and compact Aβ deposits [67]. The murine chimeric form of aducanumab cleared both diffuse and compact plaques in aged 22 month-old Tg2576 female mice in a dose-dependent manner [40]; in a separate cohort of nine month-old male and female mice treated with weekly IP injections for six months, Aβ deposits of all sizes were cleared by about 70% [40]. Two to four treatments of aducanumab in APP/PS1 mice appeared to be more efficient at the clearance of small diffuse plaques compared to larger plaques [68]. There remain important questions regarding the characteristics of plaques that are cleared earliest in these treatments.

Another model combined using an antibody with high affinity for the pyroglutamate residue at Aβ amino acid 3 (BAMB31) with a BACE1 inhibitor [69]. Aged APP/PS1 and PDAPP mice were treated weekly with IP injections for 11 weeks with one or both components of the combination therapy. Both the antibody individually and in combination with BACE1 inhibitor significantly cleared Aβ deposits [69]. Another study used a non-pharmacological combination approach in APP23 mice, combining aducanumab therapy with focused ultrasound to create microbubbles, effectively decreasing plaque-load [70]. Combination therapies need to be evaluated further for their potential to increase efficacy and translatability of various antibodies.

### Age and sex effects

Another important factor impacting the efficacy of anti-Aβ immunotherapy may be the age of the mice. One study specifically analyzed the effect of age, treating 20-month-old Tg2576 mice and 9-month-old APP/PS1 mice with antibody 2H6 IP over three months. Independent of the initial amount of Aβ and clearance of Aβ, there were increased microhemorrhages in aged mice and increased infiltration of peripheral monocytes labeled with GFP [71]. This observation suggests that models studying these therapies in younger mice may not be capturing the full scope of their effects in aged AD patients (discussed in greater detail below).

Little direct comparison of sexes has been done. The monoclonal Aβ protofibril antibody PFA-1 caused a decrease in brain levels of Aβ in 22 month-old 3xTg male mice treated following weekly IV injections for four weeks; there was a corresponding increase in plasma Aβ as measured by ELISA [36]. However, there was no change in brain Aβ levels in female mice, with a decrease in plasma Aβ40 levels. The findings also suggest a movement of at least some of the Aβ in the brain parenchyma to the blood from Aβ immunotherapy, but only in male mice in this model [36]. The differences in results between male and female mice suggest potential sex differences in the efficacy of Aβ clearance and movement. Given that sex differences have been observed in some clinical studies of Aβ immunotherapy [5], future work into the impact of sex on these mechanisms is warranted.

### Treatment timing

Preclinical studies showed that lecanemab (mAb158) and donanemab (then referred to as a “plaque specific antibody”) were able to both prevent plaque formation when administered before initial plaque development and clear existing plaques [34, 72, 73]. The mAb158 selectively cleared protofibrillar Aβ when administered IP for 13 weeks. Notably, mAb158 also significantly reduced cerebrospinal fluid levels of protofibrils, while not significantly impacting levels of monomeric Aβ in the brain [74]. This antibody has been shown to have a lasting effect: when administered via weekly IP injections for 18 weeks and then stopped for 12 weeks, the levels of soluble Aβ and insoluble Aβ, and the total number of plaques, all remained decreased [75]. Similar results were seen in APP/PS1 mice treated with aducanumab: levels of amyloid were reduced 15 weeks after cessation of treatment, but had recovered by 30 weeks [68]. Finally, a recombinant version of mAb158 modified into a bispecific format (RmAb158-scFv8D3) was able to clear protofibrils with a 10-fold lower dose following a single IV injection [55]. Together, the studies in this section of the review established that antibodies directed against Aβ are able to clear existing plaques, prevent new plaque formation, and that these effects last for a finite period of time.

### Risk of ARIA

Novel antibodies have been developed with the aim to decrease the risk of ARIA associated with anti-Aβ immunotherapy. The antibody SAR228810, targeting protofibrillar Aβ with limited Fc effector functions, was injected weekly IP in male two month old APPSL mice for 20 weeks [76]. This treatment effectively prevented plaque development, without causing microvascular alterations and inflammatory infiltrates, as compared with 3D6 [76]. Another monoclonal antibody, 5C8H5 (derived from the 4Aβ1–15 vaccine) was found to clear plaques without a significant increase in microglial activation or microhemorrhage frequency [38]. De-glycosylation of the antibody 2H6 allowed clearance of amyloid plaques and relief of cognitive impairments, with fewer vascular deposits and microhemorrhages compared with the intact 2H6 in a chronic treatment model in Tg2576 mice [77]. These studies show that the inhibition of plaque formation and clearance of existing plaques are not intrinsically tied to microvascular alterations in chronic inflammation mouse models. De-glycosylation of antibodies is an interesting approach that warrants further research.

Changes in the method of application of antibody could also address the risk of ARIA. Bypassing the vasculature was achieved by injecting a single 10 µg dose of anti-Aβ antibody directed against amino acids 1–28 into the third ventricle of 10-month-old Tg2576 mice. This single injection both reduced cerebral plaque-load, and reduced the number of IL-1β expressing microglia around plaques, all with no signs of microhemorrhage [78]. However, there remain questions regarding whether intracerebral injection of anti-Aβ antibodies clears existing plaques or prevents future deposition [79]. In addition, the practicality of direct injection of antibody into the brains of human patients presents challenges for the translation of these studies.

The precise mechanism of ARIA is not known, and there are still many unanswered questions regarding the prevention and treatment of ARIA. Several of the proposed mechanisms, including cerebral amyloid angiopathy (CAA), blood brain barrier (BBB) breakdown, and excessive neuroinflammation, will be discussed in the next sections of this review.

## Vascular dysfunction

Given the ARIA side effects of anti-amyloid immunotherapies, increasing attention is being directed to mechanisms of cerebrovascular leakage (edema) and breakage (hemorrhage). There was early evidence in mouse models that passive immunotherapy could lead to vascular breakdown: treatment with the β1 mouse monoclonal antibody, directed against Aβ amino acids 3–6, showed a doubling of hemorrhages in 21-month-old APP23 mice [39]. It was suggested that this effect could be due to the presence of existing vascular amyloid damage [39].

### Cerebral amyloid angiopathy (CAA)

A major risk factor for ARIA in humans is CAA [80]. This deposition of amyloid in the cerebrovasculature is a common co-pathology in AD brains, present in almost 50% of patients with the most advanced parenchymal Aβ deposits [81]. CAA occurs most commonly in large meningeal vessels, with amyloid accumulating first on the parenchymal side on the blood vessels (e.g., around smooth muscle cells) [81]. Compared to parenchymal amyloid deposits, CAA has a much higher proportion of Aβ40 [74, 82], through the addition of Aβ40 peptides to initial deposits of Aβ42 species and spread along affected vessels [83]. CAA amyloid also differs from parenchymal amyloid in that it includes antiparallel structures of Aβ fibrils in addition to the parallel structures [84]. The presence of CAA is associated with white matter damage and intracerebral hemorrhages [85], and on rare occasions with related inflammation (CAA-ri) [86]. Immunosuppressive therapies are successful in the treatment of CAA-ri [87].

Several studies have used mouse models that allowed specific analysis of CAA clearance. Antibody 10D5 applied directly to the brain post-craniotomy in one year old Tg2576 caused a modest decrease in CAA following a single treatment at seven days post-treatment, with a more significant attenuation of CAA levels upon chronic treatment, as measured by in vivo multiphoton imaging [88]. More recently-deposited CAA was more readily cleared than CAA that had existed for longer periods of time, as determined by an increase in the lengths of the unaffected vessels between amyloid deposits [88]. One study using 3D6 found a comparatively higher reduction in vascular amyloid compared to parenchymal plaques, with the remaining vascular amyloid appearing more fragmented [89]. Other studies assessing peripheral IP 3D6 treatment weekly showed clearance of vascular amyloid correlating with an increase in microhemorrhages [89–91]. Clearance of vascular Aβ was found to be both spatially and temporally associated with microhemorrhages [91].

There is also the possibility that passive immunotherapies could increase CAA. Tg2576 mice treated with monoclonal antibody 2286 for three months showed the expected reduction in parenchymal amyloid. However, there was also a corresponding increase in both CAA (up to four-fold) and microhemorrhages [92]. At affected vessels, there was a corresponding elevation in microglial activation [92]. The mechanisms of vascular amyloid clearance versus deposition may depend on the dose and timing regimen of antibody treatment and whether the mouse model used is susceptible to CAA formation at the ages studied.

The presence of CAA is an important factor in the outcome of Aβ immunotherapies in mouse models. Both 3D6 and 10D5 antibodies injected IP bound to vascular amyloid and loci of CAA in PDAPP mice [90, 93]. While antibodies 3D6 and 10D5 (N-terminus targeting antibodies) bound vascular amyloid, antibody 266 (which binds to the mid-terminus of Aβ) did not bind to vascular amyloid and did not cause any incidence of microhemorrhage [93]. These data suggest that antibodies targeting different epitopes of Aβ have different potential in binding and clearing vascular amyloid, just as different antibodies exhibit different abilities to clear parenchymal plaques. This finding could inform which antibodies are more likely to produce ARIA clinically.

### Blood brain barrier (BBB) integrity

Several mouse studies tested for evidence of intracerebral hemorrhages, generally through hemosiderin staining for deposited iron from red blood cells that had entered the brain. Elevated levels of hemosiderin staining after anti-amyloid therapies were observed in many studies [34, 39, 71, 76, 89–94]. The increase in hemosiderin staining was prevented through the use of deglycosylated antibody [77] or an antibody against pyroglutamate-modified Aβ [34], suggesting that hemorrhages are not an inevitable characteristic of amyloid removal. While there remain questions about the mechanisms of this vascular breakdown, there is clear evidence that it occurs in multiple mouse models.

Some mechanistic studies have focused on changes in vessel integrity. BBB breakdown can be demonstrated by the presence of fibrinogen around leptomeningeal and penetrating vessels. Fibrinogen is normally absent in the brain but accumulates around damaged blood vessels, such as those impacted by CAA [95]. Upon weekly subcutaneous treatment with 3D6 for three months, PDAPP mice showed significant increases in fibrinogen around vessels impacted by CAA and microhemorrhage [94]. This breakdown may be facilitated by an upregulation of extracellular matrix-degrading enzyme activities (matrix metalloproteinases 2, 3, and 9) after treatment from one to three months with the 2286 antibody [96]. On Gadolinium-enhanced MRI, weekly 3D6 treatment of aged PDAPP mice showed BBB breakdown at doses as low as 3 mg/kg and as early as 3 weeks after treatment initiation [97]. This disruption occurred in leptomeningeal and midline vessels affected by CAA and resolved transiently even without cessation of treatment; Prussian Blue staining indicated that these loci were associated with past microhemorrhages [97]. However, in another model, there was no significant increase of dextrans crossing the BBB into the brain three days after 3D6 injection, despite 3D6 entering the brain and accumulating around regions of CAA [98]. Overall, mouse models consistently show anti-Aβ immunotherapies causing microhemorrhage in regions of CAA.

Disruption of the BBB can lead to the infiltration of peripheral immune cells into the CNS, inducing neuroinflammation [94]. PDAPP mice treated with 3D6 chronically over one month demonstrated increased levels of astrocytes and macrophages, and was associated with a significant decrease in smooth muscle cells in CAA-afflicted vessels, a measure of decreased vascular integrity [89]. In a related study, these mice showed significant co-localization of peripheral immune cells with CAA afflicted vessels, particularly at leptomeningeal and penetrating vessels with significant microhemorrhages; the most prominent of these peripheral immune cells were monocytes [94]. More work needs to be done to define the relative contributions of various peripheral immune cells versus central immune cells to damaging neuroinflammation that occurs with anti-amyloid therapies.

### Neuroinflammation

Early post-mortem studies of individuals who had undergone active Aβ42 immunization demonstrated increased numbers and ramification of microglia [99]. Defining the aspects of this neuroinflammatory response is an important goal of mouse models of Aβ immunotherapies.

Most studies have used measures of microglial numbers, location, and morphology to draw conclusions about the neuroinflammatory state of the brain. The responses of microglia to immunotherapy change over time. Intracranial injection of the anti-Aβ antibody 6E10 into Tg2576 mice caused shifts in transcriptomic signatures of several classes of microglia within 24 h [100]. Topical application of 10D5 in APP/PS1 mice showed via in vivo brain imaging that microglia collected around plaques within one week, and the size of these microglia and the number of their processes increased [101]. Over several weeks, microglia also increased in both number and inflammatory phenotype post-treatment with multiple aducanumab doses [68]. After chronic dosing, single-cell suspensions that were analyzed by flow cytometry and RNAseq showed a significant upregulation in genes associated with microglial activation, antigen presentation, and lysosomal degradation [68] (this finding is consistent with another study that showed increases in terminally inflammatory microglia after five days of IP aducanumab treatment [102]). Thirty days after the final dose, microglia demonstrated an inability to reactivate despite the reappearance of amyloid plaques [68]. The authors hypothesized that microglia may not only be impacted by the number of doses or length of treatment, but also for extended periods of time following cessation of treatment [68]. The exact mechanisms behind these changes and the implications for patients receiving these therapies requires further investigation. For instance, it is unclear how chronic neuroinflammation would impact the clearance of amyloid deposits after immunotherapy or their reappearance after treatment cessation.

Fc-receptor activation is an important component of the immune response. Antibody 2286 increased Fc-receptor activation on microglia within one month of treatment, as measured by immunostaining, with 100-fold induction of Fc-receptor expression in the hippocampus [43]. Levels of Fc induction remained constant between one and two months, and then decreased to control levels by three months. Levels of CD45, a marker of immune cell response increased slightly in the first month, but increased over three-fold more significantly by two months of treatment [43]. The importance of the intact antibodies on microglial responses was addressed in another model using a cross of PDAPP mice with mice with GFP-fluorescent microglia, using 2-photon confocal microscopy [31]. IP injection of the antibody 3D6 into PDAPP mice increased co-localization of microglia with Aβ plaques in vivo just three days post-treatment with twice as many processes protruding from the cell body compared with untreated mice [31]. When the Fc portion of the 3D6 antibody was removed, there was no significant microglial activation as measured by total microglial numbers and numbers of processes, demonstrating the importance of the Fc-receptor in the initial microglial response [31]. Together, these two studies indicate that the initial steps of plaque clearance by microglia may be mediated by Fc-receptors. Given the limited analysis of this mechanism so far, further studies are needed to elucidate the timeline and whether these effects may vary based on the specific mouse model and antibody.

In addition to anatomical and morphological microglial changes, Aβ immunotherapy affects cytokine levels in the brain. In Tg2576 mice treated with 2286 for up to three months, there were increases (some transient, some sustained) in the pro-inflammatory markers TNF-α, IL-6, and TGF-β [103]. In 22-month-old 3xTg mice treated IV weekly for four weeks with antibody PFA-1, female mice (but not male mice) exhibited significant increases in CNS TNF-α and MCP-1 levels compared with non-immunized mice; both TNF-α and MCP-1 were found to be positively correlated with brain levels of Aβ [36]. 3D6 (but not other antibodies) increased IL-1β and TNF-α levels within one week after direct injection into the hippocampus, when strong amyloid clearance was observed [61]. However, activating microglia with IFN-γ in a locally applied model of 10D5 treatment, or inhibiting microglia with immunotoxin or minocycline, had little or no effects in amyloid clearance in APP/PS1 mice [104]. The role of cytokine involvement in promoting amyloid clearance by microglia thus remains an open question.

The efficacy of Aβ clearance by immunotherapy may depend on immune cells and macrophages from the meningeal lymphatic system. Direct injection of the monoclonal NAB61 antibody (against nitrated Aβ) into the hippocampus of Tg2576 mice resulted in mononuclear peripheral cells around amyloid-laden vessels within one week [105]. Weekly IP treatment of 5XFAD mice with aducanumab for eight weeks was less efficient at Aβ clearance in mice that had undergone ablation of the lymphatic system, which decreased levels of homeostatic microglia as defined by single-cell RNAseq [106]. Homeostatic microglia were recovered after removal of Aβ by aducanumab [106], suggesting that the regulation of glial activation by the meningeal lymphatic system affects the efficacy of antibody-mediated clearance of Aβ.

Thus, microglia are activated quickly in response to anti-Aβ immunotherapy, and based on the results of a few studies, their clearance of plaques may be mediated by Fc-receptor mechanisms. The chronic effects of anti-Aβ immunotherapy on microglia is less well-defined, but critical to an understanding of neuroinflammatory side effects of ARIA in humans, which can develop over years [107]. Mouse models are well positioned to allow the determination of how microglia respond to anti-Aβ immunotherapy temporally, and how mechanisms of plaque clearance may change over time.

## Neuronal dysfunction

Although mouse models have mainly been used to evaluate the efficiency of anti-amyloid antibodies to remove amyloid, there are many indications that these immunotherapies have neuroprotective functions. These measures are diverse, including phospho-tau accumulation, synapse activity, neuronal morphologies, and mouse behavior. In all cases, more systematic analyses across a greater number of studies are needed to understand how quickly and how well neuronal function recovers after the clearance of amyloid.

### Tau-related changes

Some studies of amyloid mouse models incorporated potential effects of anti-Aβ immunotherapy on phospho-tau, the main component of the second protein accumulation in AD brain. Direct injections of anti-Aβ antibodies (4G8 and 1560) into the hippocampus of 12-month-old 3xTg mice produced a reduction of somatodendritic tau after reducing amyloid, perhaps through increasing proteasomal activity [19]. A two month IP treatment of APP/PS1 mice with A8 antibody showed reductions in p-tau 231 as measured by both western blot and immunostains of neuritic plaques [108]. There was a reduction in total tau immunostaining (but not AT8 phospho-tau staining) observed after two weeks of scFv-h3D6 treatment [109]. On the other hand, in a study of APP/PS1 mice crossed with Tau22 mice, no changes to tau were seen despite a 70% reduction in amyloid after long-term treatment with aducanumab [110].

### Synaptic markers

These models also address whether there are long-term benefits of amyloid immunotherapy toward synaptic markers. In terms of numbers of synapses, 3D6 treatment for six months resulted in about a 20% increase in 18-month-old PDAPP mice, as measured by synaptophysin staining of hippocampal subregions [111]. Aged Tg2576 mice (18–26 months) showed a 50% increase in synapse numbers 30 days after a single topical treatment of 3D6 [112]. Two month treatment with two anti-Aβ antibodies (6G1 and an oligomeric-specific antibody) showed trends toward reducing amyloid-related synapse loss in 14-month-old Tg2576 mice [113]. There were no effects of aducanumab on synapse numbers in a three-month treatment in an APP/PS1 tau model [110], and synaptic effects in a tet-off APP transgenic mouse treated with the Ab9 antibody depended on stopping new Aβ production at the same time as the treatment [114].

### Neuronal integrity and function

Early neuroprotective effects of anti-amyloid immunotherapies are based on models of applying anti-Aβ antibodies directly to the brain and observing neurons in vivo through cranial windows. Aged (22-month-old) Tg2576 mice treated with topical aducanumab demonstrated via in vivo multiphoton imaging a fourfold increase in the number of neurites exhibiting dysregulation of calcium signaling; this aberrant response diminished over the next few weeks [115]. Worse cortical hyperactivity (as judged by calcium transients) was also observed over three months following weekly IP 3D6 administration in 12 to 17-month-old PDAPP mice [116]. This finding was replicated in Tg2576 mice treated with chronic treatment with an anti-Aβ-3-6 antibody; however, this effect occurred in the absence of amyloid clearance [116]. Thus, there is evidence of a transient induction of neuronal dysfunction in the initial stages of amyloid detection and clearance through anti-amyloid approaches.

Other measures of neuron integrity have been examined. Seven month IP treatment with an anti-Aβ antibody in APP/PS1 mice increased the neuronal complexity of newly born hippocampal neurons, as measured by neuronal branching, synaptophysin immunostaining and dendritic spine numbers [117]. Axonal degeneration was measured in APP/PS1 mice between 6 and 9 months of age, and an antibody against the N-terminus of Aβ over three weeks increased monoamine axon densities [118]. Brain atrophy was examined by MRI, and there were trends for effects of scFv-h3D6 treatment in 3xTg mice from 5 to 12 months of age [119]. As mentioned above, more systematic analyses of mouse models could identify reproducible indicators of neuronal protections.

### Mouse behavior

Many studies have looked for behavioral improvements that might accompany the removal of plaques due to immunotherapy, focusing mainly on behaviors associated with learning and memory. Treatment of Tg2576 with antibodies 2286 and 2H6 demonstrated improvement of mice in the radial arm water maze [77, 92]. APP/PS1 mice treated with 3F5 showed improvement in the Morris Water Maze (MWM) [37]. Treatment of 17–19 month old PDAPP mice with 10D5 over several months led to an improvement in the MWM [120], and aducanumab-treated APP23 mice showed improvements in an active place avoidance paradigm [70].

Other studies were less clear in demonstrating beneficial effects of immunotherapy. APP/PS1 mice treated with the 20 mg/kg A8 antibody for 8 weeks showed a strong reduction in Aβ, but despite this long time course and the use of 16 mice per condition, there was little improvement in the MWM [108]. One-year-old Tg2576 mice treated IP with BAM-10 four times over 12 days showed behavioral improvements in repeated MWM trials, but only after selection of the mice that showed an impairment before treatments [121]. Another study of Tg2576 mice showed little improvement in the MWM after months of treatment with ScFv [122]. Behavioral improvements are often difficult to measure, and it remains a target to identify consistent conditions for robust responses to anti-Aβ therapies across mouse models.

Interestingly, some early studies with passive immunotherapy produced behavioral benefits even without clearance of plaques. Upon treating Tg2576 mice for six months with the antibody NAB61, which targets a conformational epitope present in dimeric, small oligomeric, and larger Aβ deposits, mice exhibited improved spatial learning and memory without effectively clearing Aβ deposits or impacting plaque morphology or density [123]. Similarly, in another paradigm, an anti-Aβ mouse monoclonal HJ3.4 IgG antibody, recognizing the mid N-terminal of Aβ, was given IP to APP23 mice weekly for 50 days. This treatment, along with co-treatment with an LXR agonist, also improved cognitive outcomes, but did not significantly clear Aβ [124]. Similar findings were found in studies of Tg2576 mice treated with an anti-β-sheet conformation antibody (aβComAb), producing significant effects on behavioral testing without plaque clearance [125].

### Toxic Aβ species

These behavioral data suggest that early protective effects may be due less to the removal of deposited amyloid plaques and more to the neutralization of soluble toxic Aβ species [115, 126]. In studies of direct application of anti-Aβ antibodies to the brain, the measures included a small increase in the number of dendritic spines in regions distant from plaques within one hour [127], and decreased aberrant neuritic curvatures within 4 days [128]. Other studies of peripheral antibody treatments showed, in the absence of reductions in Aβ, improvements in synapse markers both near to and far from amyloid plaques [113]. Protection was also provided against neuronal damages that accrued with induction of seizures in the SwAPP model before amyloid plaques accumulated [126]. Thus, immunotherapies may be beneficial even in the absence of total amyloid clearance.

## **A role for ***APOE*** genotype**

APOE genotype has the strongest genetic impact on late onset AD. *APOE* exists in three common alleles, *APOE2*, *APOE3* and *APOE4* [129]. Compared to the most common *APOE* genotype in the US, *APOE3/3*, each *APOE4* allele decreases the age of onset of AD by about seven years, while *APOE2* alleles delay the age on onset [130]. *APOE4* individuals with late onset AD have higher levels of brain amyloid (parenchymal and CAA) [131–133]; *APOE2* individuals have lower levels of parenchymal Aβ, although, paradoxically, a higher incidence of CAA [134–136]. *APOE4* individuals have a much higher incidence of both ARIA-E and ARIA-H, with rates increased by two to five fold, across clinical studies [5]. The increased risk of ARIA in *APOE4* individuals may be related to their increased prevalence of CAA [100]. It has been recommended that *APOE4* homozygote individuals more strongly consider the risks of ARIA when deciding whether to initiate treatment [137]. Despite this strong effect of *APOE* genotype on successful outcomes of anti-amyloid treatments, there are very few mouse model studies incorporating this risk factor [138].

In one study, *APOE* knock-in mice were crossed with APP/PS1 mice and treated with IP 10D5 over three months. *APOE2*, *APOE3*, and *APOE4* mice all displayed similar clearance of parenchymal amyloid and CAA [139]. After this clearance, *APOE4* mice showed the highest levels of microglia/macrophage as measured by Iba1 and CD68 markers [139]. In another study, single cell RNA sequencing of microglia from EFAD mice (*APOE* knock-in mice crossed with 5XFAD) identified different classes of activated microglia at baseline, with significantly more terminally inflammatory microglia in both E4FAD mice and in aged mice, before treatment [102]. After 60-week-old EFAD mice were treated with daily injections of aducanumab for 5 days, there were elevated levels of microglia in various stages of activation [102]. These results were most profound in the E4FAD mice, supporting the idea that *APOE4* genotype influences microglial phenotypes following anti-Aβ immunotherapy [102]. Further understanding of how *APOE4* genotype modulates the early and late microglial responses to anti-Aβ immunotherapy is critical, particularly whether it is dependent on the higher levels of parenchymal and vascular amyloid.

## Conclusions

It has been over 25 years since Dale Schenk and colleagues first reported the effectiveness of anti-Aβ immunotherapy for clearing amyloid in preclinical mouse models [10]. The earlier work that anti-Aβ monoclonal antibodies disrupted amyloid deposits in vitro [140] raised the possibility that the presence of anti-Aβ antibodies in the brain could be a treatment for AD. Tremendous progress has been made advancing this approach and achieving clinical benefits. However, many questions remain about how Aβ immunotherapies work, questions which need to be addressed to improve effectiveness and reduce the adverse effects that result from this disease-modifying treatment. As noted throughout this review, these questions include improving the pathways of antibody entry into the CNS, identifying which amyloid types are favored in microglial phagocytosis and degradation, defining the mechanism of clearance of Aβ through the cerebrovasculature, testing whether the effects of *APOE* genotype depend on the levels of amyloid, and comparing the inflammatory pathways that are active acutely versus chronically. The study of CAA is particularly important, given its role in spontaneous and induced forms of ARIA, including the possibility of the formation of new CAA and how removal of existing CAA may lead to leakage of vessels. Mouse models also hold the promise of being able to directly compare the efficacy of different amyloid antibodies and treatment protocols. Recent clinical studies have shown that modified titration schedules of Aβ immunotherapies can decrease rates of ARIA in patients receiving donanemab [141]. These findings established that rates of ARIA can be lowered and furthers the importance of mouse studies to better develop approaches to lower ARIA rates. Expanding the study of mouse models of Aβ immunotherapies is a powerful example of research from the bench to the bedside and back to the bench.

## Data Availability

Data sharing is not applicable to this article as no datasets were generated or analyzed during the current study.

## Abbreviations

AD: Alzheimer’s Disease
Aβ: Amyloid-beta
CAA: Cerebral amyloid angiopathy
BBB: Blood brain barrier
DAM: Disease associated microglia
PS1: Presenilin-1
APP: Amyloid precursor protein
MAPT: Microtubule-associated protein tau
IP: Intraperitoneally
IV: Intravenously
